# Relationship of blood pressure status, dietary factors and serum electrolytes of in-school adolescents in Ilishan-Remo, Ogun State, Nigeria

**DOI:** 10.4314/ahs.v21i4.32

**Published:** 2021-12

**Authors:** Olutayo S Shokunbi, Ngozi A Ukangwa

**Affiliations:** Department of Biochemistry, School of Basic Medical Sciences, Babcock University, Ilishan-Remo, Ogun State, Nigeria

**Keywords:** Adolescents, dietary patterns, hypertension, table salt

## Abstract

**Background:**

Globally, rising blood pressure is of public health concern as it is a major cause of cardiovascular diseases (CVDs), and preventable death. This study accessed the relationship of blood pressure status, dietary factors and serum electrolytes among in-school adolescents in Ilishan-Remo, Ogun State, Nigeria.

**Methods:**

A cross-sectional survey of 488 secondary school students (aged 10–19 years). Blood pressures were assessed using auscultatory method and questionnaires were used to obtain food frequency and 24-hour dietary recall data. Blood samples from volunteers were used for serum sodium and potassium assays.

**Results:**

The mean systolic blood pressure (SBP) increased with age, irrespective of gender. The prevalence of elevated blood pressure and hypertension among participants were 19.3% and 10.5%, respectively, with males and females having similar pattern. Dietary factors like addition of table salt to already prepared foods, higher intake of eggs, and lower intake of vegetables were associated with the development of elevated blood pressure among the adolescents. The estimated mean dietary intakes (mg/person/day) of sodium and potassium were 2289±938.7 and 1321±603.8, respectively, with majority consuming far higher (for sodium – 80%) or far below (for potassium – 95%) recommendations. The mean serum sodium (138.0±18.3 mmolL^-1^) and potassium (3.06±1.1 mmolL^-1^) were similar across genders. A significant (p<0.05) negative relationship exists between serum potassium and SBP.

**Conclusions:**

The blood pressure status of the adolescents studied are of great concern and are somewhat negatively influenced by poor dietary and lifestyle practices. They require prompt intervention to slow down the development of CVDs in the future.

## Introduction

Adolescence involves rapid physical and emotional development^1^. Globally, hypertension is of concern to public health professionals as it is widely implicated in causing of cardiovascular disease (CVD) that could result in premature mortality^2^. As a result of multiple factors, the younger group of people are known to have a higher risk of developing cardiovascular complications from hypertension compared with those older^3^. Those that develop hypertension earlier in life have greater reduction in lifespan, if the condition is not treated^4^.

Some factors are implicated in increased prevalence of hypertension across various populations. They are unhealthy diet, smoking, physical inactivity, a family history of hypertension, and obesity. Most adolescents are usually healthy and hardly visit hospitals until when critically ill; which makes it difficult for early detection of hypertension among them^5^. Most of the risk factors related with hypertension among adolescents are quite difficult to modify as experienced during management/intervention^6^.

The prevalence of elevated blood pressure and hypertension in children and adolescents over the years has been rising, especially in the same trend with obesity. Weight loss, exercise and dietary intervention are treatment methods which require a great deal of motivation, especially on the part of the individual involved and the care provider^7^. As far as overweight and obesity are concerned, every 10 kg increase in body weight can result to 3.0 and 2.3 mm Hg increase in systolic and diastolic blood pressure, respectively^8^.

Dietary patterns are referred to as the reflection on the amount and type of foods eaten in combination and they account for the joint effects of these foods and nutrients on consumers^9^. The American Heart Association advises that individuals consume a diet containing low-fat dairy, vegetables, whole grains, fruits, lean sources of protein, including legumes, poultry, and lean meats, as well as consumes oily fish, at least twice weekly^10^. Diet has been recognized as one of the most important risk factors in the etiology of hypertension. Thus, an increased rate of hypertension and other chronic diseases, especially in developing countries have been linked to nutritional transition and food consumption patterns in recent years^11^. Research has established that the risk of stroke and other CVDs decrease with lower salt intake^12^. It has been noted that the potassium and sodium content of foods tends to be inversely related: foods with high sodium content usually have low potassium content and vice-versa^13^. In recent times, salt consumption is on the increase, especially because of its use in highly salted processed foods and food seasoning^14^. Soft drinks have also been known to be another source of caffeine, sugar, and sodium in foods. They can lead to accumulation of excess weight, especially in sedentary adolescents^15^.

Over the last one decade, several studies have been done in relation to the hypertensive status of adolescents within Nigeria^16^–^27^. Most of these researches focused on prevalence of hypertension as related to anthropometric parameters of the adolescents and only five of them have been conducted within the South-Western Nigeria; with just one done in Sagamu, Ogun State^24^. Thus, there are limited data on hypertension status of adolescents within Ogun-State, especially as related to dietary factors and serum electrolytes. Therefore, this study was designed to evaluate relationships of blood pressure status, dietary factors and serum electrolytes among in-school adolescents in Ilishan-Remo, Ogun State, Nigeria. For the purpose of this study, the non-boarding schools were selected to better represent adolescents from the study area and to minimize confounding factors.

## Methods

### Study location

The study was carried out within four secondary schools located in Ilishan-Remo town, under Ikenne Local Government Area of Ogun State, Nigeria. Ilishan-Remo is located within latitude 6.8932 North and Longitude 3.7105 East in the rainforest zone of Nigeria. Relative to land size, it is the fourth among thirty-three towns within Remo Division of this State. Most of the inhabitants of the town are farmers and traders^28^. It is the town hosting Babcock University.

### Study design and sampling

The study employed a multi-stage sampling technique. Four non-boarding secondary schools (2 public and 2 private) in Ilishan-Remo, Ogun State were purposively selected. The schools were stratified into junior and senior sections and 488 adolescents were finally selected (proportionate to the size of each school) using simple random technique. Only students within the ages of 10 to 19 years were included in the study. The study was carried out from October to December, 2018.

The participants' blood pressure measurements were taken along with an interviewer-administered 24-hour dietary recall. Then, a well-structured questionnaire, including socio-demographic characteristics of the participants and food frequency was self-administered. Last, about 5 mL blood sample was drawn from each volunteer, for serum electrolyte analyses.

### Inclusion and exclusion criteria

All students within the ages of 10 to 19 years were included in the study and those below or above these ages were excluded. Those that did not give their consent were also excluded. All participants recruited were apparently healthy and those on antimalarial, antibiotics, and antihypertensive drugs were excluded.

### Blood pressure measurement

The systolic and diastolic blood pressures were measured in accordance with the clinical practice guideline for screening blood pressure in children and adolescents^29^. Blood pressure (BP) was measured by licensed medical personnel using the auscultatory method. This involved the use of a stethoscope and a manual mercury sphygmomanometer with appropriate cuff size with a bladder length of about 80% to 100% of the circumference of the arm and a width of at least 40%. Measurements were done using the right upper arm after the volunteer had been allowed to seat quietly for up to 5 minutes, with feet being uncrossed on the floor. The first (phase I Korotkoff) and last (phase V Korotkoff) audible sounds were taken as systolic blood pressure (SBP) and diastolic blood pressure (DBP), respectively. The measurements were taken three times in a single visit, with allowance of at least 3–5 minutes in between the three readings, and a mean of the readings was reported as the individual's BP. The BP data was collected from participants between 7 am and 9 am (before break-time) of each day of study.

### Determination of blood pressure status

The clinical practice guideline for screening of blood pressure in children and adolescents^29^, was used to evaluate the BP status of the adolescents. For participants less than 13 years, normal BP was indicated as SBP and/or DBP <90^th^ percentile for sex, age, and height. Elevated BP was indicated as SBP and/or DBP ≥90^th^ percentile and <95^th^ percentile or 120/80 mm Hg to <95^th^ percentile (whichever is lower) for sex, age, and height. Stage 1 hypertension (HTN) was indicated as SBP and/or DBP ≥95^th^ percentile to <95^th^ percentile + 12 mm Hg, for sex, age, and height or 130/80 to 139/89 mm Hg (whichever is lower). Stage 2 HTN was indicated as SBP and/or DBP ≥95^th^ percentile + 12 mm Hg or ≥140/90 mm Hg (whichever is lower), for sex, age, and height. For participants that were 13 years and above, the following parameters were used for their classification: Normal BP: < 120/< 80 mm Hg; Elevated BP: 120/< 80 to 129/< 80 mm Hg; Stage 1 HTN: 130/80 to 139/89 mm Hg; Stage 2 HTN: ≥ 140/90 mm Hg.

### Estimation of mean dietary intake of sodium and potassium

A WHO standard 24-hr dietary recall questionnaire was used for the assessment of daily sodium and potassium intakes of the participants. The participants were interviewed by trained research assistants using some food models to assist them in recalling the foods, fruits, and drinks consumed within the last 24-hours. The gram equivalents of the various food items eaten were used in computing the participants mean daily sodium and potassium intakes using a standard nutrient calculator software built with Java programming language (unpublished).

### Blood collection and serum electrolyte analysis

Five Milliliter of venous blood sample was drawn from each volunteer, for serum electrolytes analyses, by licensed medical personnel. The serum was separated from the red cells by spinning at 3,000 g for 10 mins. The serum was transferred into micro tubes and stored at -4 ºC until time for analysis. The serum electrolyte analyses were determined by enzymatic method using standard test kit from Teco Diagnostics (California, USA.). The analyses were done according to manufacturer's instructions.

### Ethical consideration

Participants who volunteered to partake in the study were carefully chosen from four main secondary schools in Ilishan-Remo. Prior to data collection, ethical approval (Number BUHREC622/18) was gotten from Babcock University Health Research Ethics Committee (BUHREC), Ilishan-Remo. Permission to carry out the study was also taken from the Local Educational Authority and management of the secondary schools. Certificate of research approval was also further issued from the Ogun State Ministry of Education and Ministry of Health. Written informed consent of the parent of each study participant was obtained, thereafter which written informed consent of each participant was also obtained, before being enrolled in the study. Confidentiality of the participants' data was maintained throughout the study and reporting stages.

### Data Analysis

Data were evaluated using statistical package for social sciences (SPSS) version 22.0 and Microsoft Excel (2017). Continuous variables were presented as means and standard deviations, while categorical variables were presented as percentage proportions. In addition to descriptive statistics, Multiple (linear) Regression was used to find the relationship between variables and the confidence level was set at p < 0.05.

## Results

### Blood pressure pattern of participants

The mean systolic blood pressure (SBP) and diastolic blood pressure (DBP) of the adolescents that participated in this study are as presented in Table 1. The mean SBP and DBP for male participants were 110.6 ± 15.1 and 71.2 ± 11.7 mm Hg, respectively. On the other hand, the mean SBP and DBP for female were 110.6 ± 12.8 and 72.4 ± 10.7 mm Hg, respectively. The overall mean SBP and DBP were 110.6 ± 13.7 and 71.9 ± 11.0 mm Hg, respectively. For male participants, those that were of 18 years age category had the highest mean SBP and DBP of 129.8 ±12.9 and 85.3 ± 8.3 mm Hg, respectively. Among the female participants, those within the 13 years age category had the highest mean SBP (118.8 ± 13 mm Hg) and those within 17 years age category had the highest mean DBP (79.3 ± 8.4 mm Hg). Overall, the mean SBP and DBP were highest within participant of 18 years category, which were 122.9 ± 12.4 and 82.7 ± 8.4 mm Hg, respectively. There was an apparent increasing linear trend in SBP for males but a non-linear trend in SBP for females. The overall blood pressure levels showed a gradual increase in mean SBP and DBP across the age categories (Table 1).

**Table 1:** Blood pressure level of participants based on age and gender

Age (year)	Male			Female			Overall		

	N	Mean SBP (mm Hg)	Mean DBP (mm Hg)	N	Mean SBP (mm Hg)	Mean DBP (mm Hg)	N	Mean SBP (mm Hg)	Mean DBP (mm Hg)
10	1	100.0	70.0	8	95.9±9.5	63.8±7.4	9	96.3±9.0	64.4±7.2
11	14	103.9±13.5	66.2±10.1	17	98.5±11.9	65.4±11.9	31	100.9±12.7	65.8±10.9
12	20	105.1±13.6	67.0±8.6	27	104.0±12.3	67.8±10.7	47	104.5±12.7	67.5±9.8
13	29	103.1±9.9	66.0±9.4	46	118.8±13.0	71.3±9.8	75	108.4±12.6	69.3±9.9
14	30	112.1±11.1	74.0±12.8	74	111.2±11.5	73.7±9.6	104	111.5±11.4	73.9±10.5
15	32	112.0±16.1	68.0±13.1	70	112.0±12.7	72.9±11.3	102	112.2±13.8	71.5±12.0
16	28	114.1±13.5	74.0±9.2	50	114.0±12.4	74.1±11.0	78	114.1±12.7	74.4±10.3
17	13	118.5±14.5	77.7±9.9	19	116.0±10.9	79.3±8.4	32	117.0±12.3	78.6±8.9
18	7	129.8±12.9	85.3±8.3	3	106.7±5.7	76.7±5.7	10	122.9±12.4	82.7±8.4
Total	174	110.6±15.1	71.2±11.7	314	110.6±12.8	72.4±10.7	488	110.6±13.7	71.9±11.0

DBP – diastolic blood pressure; SBP – systolic blood pressure.

The relationship of gender and age with the SBP of the participants was explored using linear regression. The regression analysis showed a β coefficient of 0.336 for age, which is statistically significant (p < 0.05). On another hand, the β coefficient for gender was 0.003 and had no significant relationship (p > 0.05) with SBP.

### Prevalence of elevated blood pressure and hypertension among participants

Table 2 indicates the overall distribution of blood pressure status among participants involved in this study. The SBP and DBP were being used to categorize them. Generally, 19.3% of these adolescents were found to have elevated blood pressure, while up to 10.5% of them fell within the hypertensive category, with only 3.9% in the stage 1 category. The table further shows a disaggregated distribution of their blood pressure status based on their gender and age. When disaggregated, about 71.3% of males were normotensive, 20.7% had elevated blood pressure and 8.0% were hypertensive. Among the female participants, 69.7% were within the normotensive category, 18.5% had the elevated blood pressure status, while 11.8% were of hypertensive status. Participants of age 10 years category had the highest percentage (88.9%) of normotensives, while those of ages 14 years (27.9%) and 18 years (20.0%) had the highest percentages of those with elevated blood pressure and those hypertensive, respectively.

**Table 2:** Distribution of blood pressure status of participants based on gender and age

Category	Normotensive	Elevated blood pressure	Hypertensive	Total
	n=343 (70.3)	n=94 (19.3)	n=51 (10.5)#	n=488 (100)
Gender	**Distribution of blood pressure status based on gender**			
Male	124 (71.3)	36 (20.7)	14 (8.0)	174 (100)
Female	219 (69.7)	58 (18.5)	37 (11.8)	314 (100)
Age (year)	**Distribution of blood pressure status based on age**			
10	8 (88.9)	1 (11.1)	0 (0)	9 (100)
11	26 (83.9)	4 (12.9)	1 (3.2)	31 (100)
12	36 (76.6)	9 (19.1)	2 (4.3)	47 (100)
13	54 (72.0)	15 (20.0)	6 (8.0)	75 (100)
14	63 (60.6)	29 (27.9)	12 (11.5)	104 (100)
15	69 (67.6)	19 (18.6)	14 (13.7)	102 (100)
16	58 (74.4)	10 (12.8)	10 (12.8)	78 (100)
17	22 (68.8)	6 (18.8)	4 (12.5)	32 (100)
18	7 (70.0)	1 (10.0)	2 (20.0)	10 (100)
Total	343 (70.3)	94 (19.3)	51 (10.5)	488 (100)

Note: values represent number of participants (percentage within gender or age category)

# 3.9% of this group are of stage 1 hypertension and 6.6% are of stage 2 hypertension category.

### Dietary factors and blood pressure status

Table 3 shows the proportion of the participants that usually consume some food items or food groups, four to six times weekly, as related with their blood pressure status. It was observed that 58.1% of participants that were hypertensive consumed eggs 4–6 times weekly. Fruits (31.4%) and vegetables (36.2%) were also less consumed by participants categorized as hypertensive. Carbonated drinks (19.0%) and puff-puff (deep fried dough made from refined white flour, sugar, salt, nutmeg and yeast) (48.6%) were found to be relatively less consumed by this same category of participants. Other food items or groups probed were consumed in somewhat similar patters among the three categories of participants.

**Table 3:** Consumption pattern (4–6 times weekly) of some food items or groups by participants as related with their blood pressure status

Food item/group	Normotensive n=343 (70.3%)	Elevated blood pressure n=94 (19.3%)	Hypertensive n=51 (10.5%)
Carbonated drinks	22.4	26.4	19.0
Puff-puff (deep fried dough)	58.8	60.6	48.6
Eggs	50.8	50.8	58.1
Fruits	25.9	28.5	31.4
Vegetables	54.2	56.5	36.2
Beef	30.5	23.3	32.3

Values represent percentage of participants that consumed the food item or food group within the blood pressure category.

**Figure 1:**
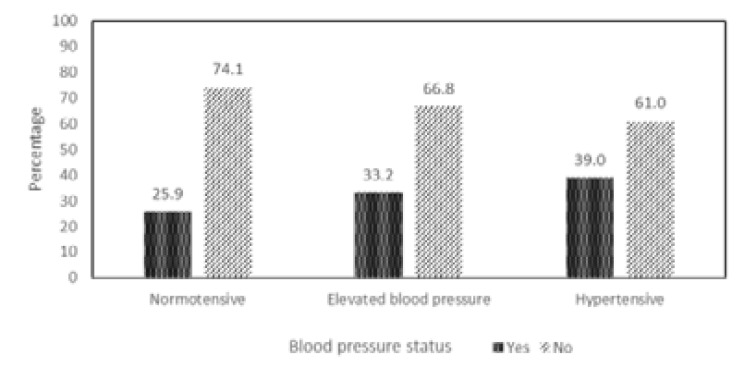
Percentage of participants who add table salt to already prepared food in relation to blood pressure status

Figure 1 represents bar charts of adolescents who usually add or do not add table salt to their already prepared foods, just before eating; based on their blood pressure status. About 39% of hypertensive adolescents add table salt to their already prepared foods. There is an apparent increase in blood pressure based on addition of table salt to already prepared foods intake. However, a Chi-square test showed no significant relationship between blood pressure status and addition of table salt to already prepared foods just before eating.

Table 4 represents the estimated average daily intakes of sodium and potassium by participants in this study. It was observed that the mean daily intake of sodium by participants (2289.2 ± 938.4 mg/person/day) was far above the recommended daily intake for this mineral. On the other hand, the mean daily intake of potassium by the participants (1328.6 ± 603.8 mg/person/day) was also noted to be far below standard recommendation. The mean daily intake of sodium for male participants (2404.2 ± 902.0 mg/person/day) was slightly higher than that of female participants (2225.3 ± 971.1 mg/person/day). The mean daily intake of potassium was with similar trend between the two groups, with males having relatively higher intakes. It was also noted that majority (about 83% and 78% of male and female participants, respectively) consumed sodium above recommendation. However, on the mean daily intake of potassium, up to 95% of both male and female participants consumed below recommendation.

**Table 4:** Estimated mean daily sodium and potassium intakes by participants

Mineral	Mean ± SD		Recommended Intake	UL
	mg/person/day			
Sodium	2289.2 ± 938.4 (198.1, 5909.3)^#^		1200–1500^a^^#^	ND^a^
Potassium	1328.6 ± 603.8 (205.3, 3878.0)^#^		2300–3000^a^^#^	ND^a^
	**Estimated mineral intakes based on gender**			
	Male	Female		
	mg/person/day			
Sodium	2404.2 ± 902.0 (9.2, 7.9, 82.9)*	2225.3 ± 971.1 (14.5, 8.0, 77.5)*	1200–1500^a^^#^	ND^a^
Potassium	1384.1 ± 971.6 (94.7, 3.9, 1.3)*	1298 ± 588 (96.4, 0, 3.6)*	2300–3000^a^^#^	ND^a^

^#^ – Values represent mean ± SD (min, max)

* Values represent mean ± SD (% consuming below recommendation, % consuming within recommendation, % consuming aove recommendation)

UL – Tolerable Upper Intake Level;

ND – no data; ^#^ - Adequate Intake (AI).

a National Academies of Sciences, Engineering, and Medicine^30^

### Serum sodium and potassium of participants

The mean serum sodium (mmol L^−1^) ranged from 137.6 ± 19.3 in females to 138.4 ± 16.9 in males, whereas mean serum potassium (mmol L^−1^) ranged from 3.6 ± 1.1 in females to 3.7 ± 1.3 in males. There was no significant difference (p > 0.05) in the serum levels of the two electrolytes, when compared across gender. A linear regression analysis of serum electrolytes with the SBP and DBP of participants showed beta values of 0.087 (p = 0.25) and 0.04 (p = 0.65), respectively for Na^+^ and beta values of -0.178 (p = 0.03) and -0.166 (p = 0.02), respectively for K^+^. This showed that K^+^ is negatively and significantly (p < 0.05) associated with SBP and DBP, whereas Na^+^ is not significantly associated with them.

## Discussion

Adolescent hypertension is becoming a cause for concern in the global community^2^. Risk factors including dietary patterns, obesity, and family history have been identified as the causes of this emerging epidemic^31^. This study investigated the prevalence of elevated blood pressure (BP) and hypertension among adolescents within secondary schools in Ilishan-Remo, Ogun State. It further evaluated the relationships of some dietary factors and serum sodium and potassium with the BP status of the adolescents. Both genders (male and female) were represented in this study; however, more females volunteered (as randomly selected) in this study than the males. This could be due to the relative distribution of the genders within the schools being studied.

This results showed an increase in mean systolic blood pressure (SBP) and diastolic blood pressure (DBP) across the ages for both genders (Table 1). Similar study carried out by Akor et al.^32^, observed that SBP and DBP increases across the ages. Studies have shown that under normal physiological conditions, as growth and body development progresses, BP values usually rise between ages 13 and 18 years^33^. The report from this study is also in agreement with a study in Jordan, conducted by Jaddou and colleagues^34^, which reported that SBP and DBP values of adolescent students increase as they grow older. Indeed, adolescence is a crucial life stage of humans with remarkably rapid growth rate^35^.

Considering the regression of age and gender with SBP, there was a significant positive relationship (p < 0.05) of age with SBP, but there was no significant (p > 0.05) association between gender and SBP. This showed that increase in age led to an increase in SBP. This was similarly reported by Akor et al.^32^, where there was a significant increase of SBP with age. However, this outcome is contrary to the report of a research done in Nnewi^25^, where there was a positive relationship between gender and SBP of the children. The trend shown in our result is clearly indicated in the disaggregated data presented in Table 2 on the BP status of participants in relation to age and gender. Males and females had similar proportions with the same BP status. There were no clear reason(s) why the males have apparently increasing linear trend in SBP, unlike among the females; though there are speculations on hormonal interplays, which will require further study to establish.

From this study, the prevalence of hypertension (10.5%) and elevated BP (19.3%) are somewhat like the outcome of study carried out on some adolescents in Lagos State, Nigeria by Oduwole et al.^21^, who reported the prevalence of 12.1% and 16.0% for hypertension and elevated blood pressure, respectively (Table 2). A similar study carried out in Ghana by Abeena36 showed a prevalence of 9.8% and 24.8% of hypertension and elevated blood pressure, respectively among the adolescents studied. Compared with previous report of Oyewole and Oritogun^24^, who reported 0–10.5% and 0–2.9% for adolescent females and males (in Sagamu, Ogun State) with pre-hypertension, respectively; our results of 18.5% and 20.7% for females and males with elevated blood pressure, respectively are quite disturbing and deserve urgent attention. On another hand, participants of ages 12 (19.1%), 13 (20%), and 14 (27.9%) years were at greatest risk of elevated blood pressure, whereas those of ages 15 (13.7%), 16 (12.8%), and 18 (20%) years were at greatest risk of hypertension (Table 2); as they had the top three highest proportions of their group members affected. This calls for further attention on the dietary patterns and lifestyles of the adolescents in these age categories, in an effort to forestall future challenges.

The relatively higher percentage (58.1%) of the adolescents in the hypertensive category consuming eggs and lowest percentage (36.2%) consuming vegetable, four to six times weekly, raise some concerns on the dietary pattern of participants with this BP status (Table 3). The carbonated drinks and puff-puff (deep fried dough) that were less consumed by hypertensive participants could give complementary positive effect on their BP status. The consumption of puff-puff (60.6%) and carbonated drinks (26.4%), four to six times weekly, by very high proportion of participants with elevated BP, were the main dietary factors of negative implications on their BP status. High consumption of fries and carbonated drinks can result in weight gain and may also increase triglycerides level. All these may contribute to future risk factors in the etiology of cardiovascular diseases9. Another major culprit dietary factor is the addition of table salt to already prepared foods (Figure 1).

The intake of high amount of sodium through foods is known to be associated with hypertension that is a leading risk factor for cardiovascular diseases (CVDs) like stroke and heart disease^37^. Addition of table salt to already prepared food before eating would definitely increase sodium intake and related risks. The trend observed among the participants showed clearly that the higher the proportion (of the blood pressure status category) that added table salt to already prepared food, the greater the percentage with hypertension. However, the outcome of the Chi-square test makes this a weak dietary factor implicated in the risk of developing hypertension. This dietary practice needs to be addressed among the adolescent to slow down or reverse their worsening BP status.

The average daily intakes of sodium and potassium by the volunteers in this study are of serious concern, as they were far higher and far lower than recommendations, respectively (Table 4). This outcome could be due to higher intakes of processed foods by these adolescents. The trend of higher dietary sodium and lower potassium intakes has also been reported among adult population studied within Ogun State and Federal Capital Territory, Abuja in Nigeria^38^. Increased dietary intake of foods rich in potassium is linked with lower BP and could be of help in the reduction of increased BP among adolescents^39^. Thus, it is needful to devise strategies to encourage the adolescents to increase their consumptions of fruits, vegetables and other foods rich in potassium as they reduce sodium intake, thereby limiting their risks of developing or retaining hypertension and CVDs.

The mean serum sodium and potassium of the participants were within normal ranges, which signifies their relative safety. These indices are not quite indicative of their current hypertension status and thus, should not be singly relied upon. Rather, other prevailing data should be holistically considered to determine the best management scheme to adopt for the adolescents. The serum potassium was notable as a better predictor of the SBP of the adolescents – a decrease in serum potassium would yield a corresponding increase in SBP.

## Conclusion

In summary, the mean SBP increases with age, irrespective of gender. The relatively high prevalence of elevated BP and hypertension among adolescents in Ilishan-Remo, Ogun State is of great concern, with similar trend across both genders; especially among those of ages 14 to 18 years. Higher intakes of eggs with lower intakes of vegetables have been implicated among the affected adolescents. Furthermore, addition of table salt to already prepared foods along with higher dietary intake of sodium and lower intake of potassium have been the other dietary factors implicated in the study. In view of these outcomes, it is strongly recommended that prompt focused attention should be given to these adolescents to encourage them on dietary and lifestyle modifications useful to forestall future challenges related to hypertension and by extension CVDs among the population. Similar public health enlightenment should be extended to other adolescents within Nigeria.

## Limitations

The limitations of this study includes the measurement of BP at a single visit, though a mean of three readings were reported and some epidemiological studies^24^,^40^ have also adopted similar strategy. Thus, caution should be taken in the interpretation of the findings reported. Limited time and available funding for the study have also been of constraints on the number of adolescents that could be incorporated.
